# A randomized half-body, double blind, controlled trial on the effects of a pH-modified moisturizer vs. standard moisturizer in mild to moderate atopic dermatitis^☆^^☆☆^

**DOI:** 10.1016/j.abd.2019.11.007

**Published:** 2020-03-21

**Authors:** Siew Wen Goh, Adawiyah Jamil, Nazarudin Safian, Norazirah Md Nor, Norliza Muhammad, Nur Liyana Saharudin

**Affiliations:** a Dermatology Unit, Medical Department, University Kebangsaan Malaysia Medical Centre, Kuala Lumpur, Malaysia; b Department of Medicine, University Kebangsaan Malaysia Medical Center, Kuala Lumpur, Malaysia; c Department of Community Health, Faculty of Medicine, University Kebangsaan Malaysia Medical Centre, Kuala Lumpur, Malaysia; d Department of Pharmacology, Faculty of Medicine, University Kebangsaan Malaysia, Kuala Lumpur, Malaysia; e Department of Pharmacy, University Kebangsaan Malaysia Medical Centre, Kuala Lumpur, Malaysia; f Dermatology Unit, Department of Medicine, University Kebangsaan Malaysia Medical Center, Kuala Lumpur, Malaysia

**Keywords:** Atopic, dermatitis, Eczema, Emollients

## Abstract

**Background:**

Higher skin pH in atopic dermatitis contributes to impaired epidermal barrier. A moisturizer compatible with physiological pH could improve atopic dermatitis.

**Objective:**

To determine the effect of a physiologically compatible pH moisturizer in atopic dermatitis.

**Methods:**

A randomized half body, double blind, controlled trial involving patients with stable atopic dermatitis was performed. pH-modified moisturizer and standard moisturizer were applied to half body for 6 weeks.

**Results:**

A total of 6 (16.7%) males and 30 (83.3%) females participated. Skin pH reductions from week 0, week 2 and 6 were significant at the forearms (5.315 [0.98] to 4.85 [0.54] to 5.04 [0.78], *p* = 0.02) and abdomen (5.25 [1.01], 4.82 [0.64], 5.01 [0.59], *p* = 0.00) but not at the shins (5.01 [0.80], 4.76 [0.49], 4.85 [0.79], *p* = 0.09) with pH-modified moisturizer. Transepidermal water loss (TEWL) at the forearms decreased (4.60 [2.55] to 3.70 [3.10] to 3.00 [3.55], *p* = 0.00), abdomen (3.90 [2.90] to 2.40 [3.45] to 2.70 [2.25], *p* = 0.046). SCORAD improved from 14.1 ± 12.75 to 10.5 ± 13.25 to 7 ± 12.25, *p* = 0.00. In standard moisturizer group, pH reductions were significant at the forearms (5.29 [0.94] to 4.84 [0.55] to 5.02 [0.70], *p* = 0.00) and abdomen (5.25 [1.09], 4.91 [0.63], 5.12 [0.66], *p* = 0.00). TEWL at the forearm were (4.80 [2.95], 4.10 [2.15], 4.60 [3.40], *p* = 0.67), shins (3.80 [1.40], 3.50 [2.35], 4.00 [2.50], *p* = 0.91) and abdomen (3.70 [2.45], 4.10 [3.60], 3.40 [2.95], *p* = 0.80). SCORAD improved from 14.2 ± 9.1 to 10.9 ± 10.65 to 10.5 ± 11, *p* = 0.00. Reduction in pH was observed with both moisturizers while TEWL significantly improved with pH-modified moisturizer. pH-modified moisturizer resulted in greater pH, TEWL and SCORAD improvements however the differences were not significant from standard moisturizer.

**Study limitation:**

Skin hydration was not evaluated.

**Conclusion:**

Moisturization is beneficial for atopic dermatitis; use of physiologically compatible pH moisturizer is promising.

## Introduction

Atopic dermatitis (AD) is a chronically relapsing inflammatory skin disease with increasing prevalence, affecting 0.2–24.6% of children worldwide.^1^ The estimated prevalence in our country was 12.6% with an increase of 0.49% yearly.^1^ Pathophysiology of AD involves complex interactions between genetic factors, the immune system and the environment. Elevation of skin pH is an important pathological component that cause increased proteases activity and inhibition of lipid lamellae synthesis, leading to breakdown of the epidermal barrier.^2^ Normal adults and adolescents have skin pH of 4–5.^3^ Patient with AD has higher pH values in lesional and non lesional skin compared to healthy population.^3^

Physiologic acidic skin pH plays an important role in preserving integrity of the epidermal barrier; it has yet to be adequately applied as the core concept in AD management. Skin acidification in murine models improved epidermal integrity, transepidermal water loss (TEWL), accelerated barrier recovery^4^ and prevented AD.^2^, ^5^, ^6^ It prevented emergence of oxazolone-induced AD, reduced Th2-dominant inflammation, normalized expression of antimicrobial peptides and inhibited generation of cytokines.^5^ Skin barrier and permeability function in normal mice improved with increased activities of β-glucocerebrosidase and sphingomyelinase, and reduction in serine protease dependent degradation of desmogleins.^4^ Application of the murine skin acidification concept in restoration of physiological skin acidity in AD and its effect on disease severity still requires further investigation.

Our study aimed to determine the effect of pH 4.5 moisturizer in restoration of epidermal barrier in AD. A moisturizer with pH that is more compatible with physiological pH could improve the skin pH in AD and subsequently its disease severity.

## Methods

A randomized half-body, double-blind, controlled study comparing a pH-modified moisturizer with a standard commercial moisturizer was performed. Inclusion criteria were patients aged 12–65 years diagnosed with AD according to the Hanifin–Radjka Criteria,^7^ and stable mild to moderate AD for 1 month prior to recruitment. Exclusion criteria were recurrent infections, >1% body surface area with skin erosions, known allergy or irritation reaction to aqueous cream, glycerine, methyl paraben or propyl paraben, change in treatment regime 4 weeks before recruitment, pregnancy and breast feeding.

Half body randomization was performed using sequentially numbered, opaque sealed envelopes (SNOSE). Investigators and patients were blinded throughout the study. Patients were instructed to apply one moisturizer on one side of the body from the neck below and one moisturizer on the other side twice a day for 6 weeks. All other topical and oral treatments the patients were using prior to study enrollment except moisturizers were continued.

Measurements of skin pH and transepidermal water loss (TEWL) were performed at baseline (week 0), week 2 and week 6 at 6 pre-determined sites, bilateral forearms, right and left side of abdomen and bilateral anterior shins. Patients rested for at least 20 min at 22 °C in relative humidity of 55–60%. Measurements were performed at least 5 h after moisturizer and other topical therapy application. Skin pH was assessed by Hanna Instruments H199181. Transepidermal water loss was assessed by Tewameter TM300. Three readings were taken per assessment and an average was obtained. A modified objective SCORing Atopic Dermatitis (SCORAD) was used to determine disease severity. The A component was doubled in the calculation of the final score. The assessment of itch and sleeplessness in component C was excluded as it was difficult to differentiate the severity of itch on each side of the body and impossible to determine the effect of symptoms from half of the body to accounts for sleeplessness. The modified objective SCORAD was calculated as 2 (*A*/5) + 7*B*/2. Score of <15 was considered mild, 15–40 was moderate, while >40 was severe.^8^ Itch was evaluated using a visual analog score (VAS) of 0–10 with 0 representing no itch, and 10 representing most severe itch. The Dermatology Life Quality Index Questionnaire (DLQI)^9^ were used to determine the disease effect on the patients’ quality of life. SCORAD, itch score and DLQI were determined at baseline (week 0), week 2 and at week 6.

The standard commercial moisturizer used was aqueous cream manufactured by KCK Pharmaceutical Industries Sdn Bhd. The ingredients include 9% emulsifying wax, 6% liquid paraffin, 15% white soft paraffin, 0.15% methyl paraben and 0.08% propyl paraben. The cream has pH of 7.32–7.58 measured by LAQUA pH meter by HORIBA Scientific. The pH of this moisturizer was modified to achieve the desired pH of 4.5 by adding citric acid 5%. Citric acid was chosen as it was easily available. Citric acid forms a small component of the natural moisturizing factors and topical application has been shown to be beneficial in ichthyiosis, increased epidermal thickness and increased dermal glycosaminoglycan.^10^, ^11^ The pH-modified cream remained stable over 3 months at room temperature without changes in color, smell, texture, consistency and pH. Both the standard commercial moisturizer cream and pH-modified cream have similar color, smell, texture and consistency. The study was approved by Research Ethics Committee, the National University of Malaysia research code FF-2018-058. Funding was obtained from the Fundamental Research Grant, National University of Malaysia with the same research code. Sample size was calculated with effect size of 0.2 and standard deviation of the outcome of 0.18 based on the results of Danby et al.^12^ Two-tailed alpha level was set at 0.05, beta level 0.2. Using the formula (1/*q*1 + 1/*q*0) (*Zα* + *Zβ*)^2^/(*E*/*S*)^2^, the sample size was 36 with consideration of 20% drop out rate.^13^ SPSS statistical software was used for statistical analyses. Where there were more than 2 variables compared, *p*-values were obtained using Friedman test. Wilcoxon test was used to determine changes between baseline, week 2 and week 6. Mann–Whitney test was used to analyze the differences between standard moisturizer and pH-modified moisturizer groups; *p*-value <0.05 was considered significant.

## Results

Thirty six patients with AD participated in the study. Thirty patients were females while 6 were males. The age ranged from 12 to 64 years while the median age was 24.5 (28.7) years old. There were 20 (55.6%) Malays, 9 (25%) Chinese, 5 (13.9%) Indians and 2 (5.5%) of other ethnicities. Twenty-one (58.3%) patients had allergic rhinitis or sinusitis, 12 (33.3%) had allergic conjunctivitis and food allergy respectively, and 29 (80.6%) had positive allergy test. Thirty (83.3%) patients were on moisturizers and 31 (86.1%) on topical steroid application, 15 (41.7%) of them required antihistamine. The types of moisturizers used by the patients were: aqueous cream 13 (36.1%), aqueous cream with glycerin 12 (33.3%), paraffin 3 (8.3%), others 2 (5.6%) i.e. 1 oatmeal based and 1 urea based moisturizer while 6 (16.7%) not using any moisturizer. Baseline disease severity assessments revealed modified objective SCORAD median value of 14.3 (10.7), body surface area (BSA) was 1.0 (1.5), itch score was 4 (3) and DLQI was 7.5 (5.75). Characteristics of the study population are summarized in table 1.

**Table 1:** Characteristics of the study population.

Characteristics	*n* (%) or median (IQR)
*Age, years*	24.5 (28.7)

*Gender*	
Male	6 (16.7%)
Female	30 (83.3%)

*Ethnicity*	
Malay	20 (55.6%)
Chinese	9 (25%)
Indian	5 (13.9%)
Others	2 (5.5%)

*Co-morbidities*	
Allergic rhinitis/sinusitis	21 (58.3%)
Bronchial asthma	9 (25%)
Urticaria/angioedema	11 (30.5%)
Allergic conjunctivitis	12 (33.3%)
Food allergy	12 (33.3%)
Drug allergy	5 (13.9%)
Positive pick/Patch test/serum IgE	29 (80.6%)

*Treatment*	
Moisturizer	30 (83.3%)
Topical steroids	31 (86.1%)
Systemic immunosuppressant	0 (0%)
Antihistimine	15 (41.7%)

*Type of moisturizer*	
Aqueous cream	13 (36.1%)
Aqueous cream with glycerine	12 (33.3%)
Paraffin	3 (8.3%)
Others	2 (5.6%)
No moisturizer	6 (16.7%)

*Disease severity*	
Modified objective SCORAD	14.3 (10.7)
BSA	1.0 (1.5)
DLQI	7.5 (5.75)
Itch score	4 (3)

### pH-modified moisturizer

Skin pH decreased from week 0 to week 6 at all sites, greater reduction was observed at week 2. The values increased slightly from week 2 to week 6 but remained lower than baseline (Table 2). The pH reductions from week 0 to week 6 were significant at the forearms 5.315 (0.98) at week 0, 4.85 (0.54) at week 2, 5.04 (0.78) at week 6, *p* = 0.024 and abdomen 5.25 (1.01) at week 0, 4.82 (0.64) at week 2, 5.01 (0.59) at week 6, *p* = 0.000 but not at the shins 5.01 (0.80) at week 0, 4.76 (0.49) at week 2, 4.85 (0.79) at week 6, *p* = 0.088. TEWL decreased from week 0 to week 6 at all sites. TEWL significantly decreased from 4.60 (2.55) at week 0 to 3.70 (3.10) at week 2 and 3.00 (3.55) at week 6 with *p* = 0.00 at the forearms (Table 2). At the abdomen, TEWL decreased from 3.90 (2.90) at week 0 to 2.40 (3.45) at week 2 but increased slightly to 2.70 (2.25) at week 6, *p* = 0.05. The changes observed over the shins were not significant, TEWL at week 0 was 3.70 (2.30), 3.50 (2.25) at week 2 and 3.50 (2.85) at week 6, *p* = 0.10. The changes in skin pH and TEWL at the pH modified moisturizer application sites are presented in table 2. SCORAD improved significantly, median SCORAD was 14.1 (12.75) at week 0, 10.5 (13.25) at week 2 and 7 (12.65) at week 6, *p* = 0.00 (Table 3).

**Table 2:** pH and TEWL values at weeks 0, 2 and 6 at pH-modified moisturizer and standard moisturizer application sites.

Parameter	Site	Week	pH-modified moisturizer	Standard moisturizer	*p*-Value
pH	Forearm	0	5.32 (0.98)	5.29 (0.94)	0.90
		2	4.85 (0.54)	4.84 (0.55)	
		6	5.04 (0.78)	5.02 (0.70)^b^	
	Abdomen	0	5.25 (1.01)	5.25 (1.09)	0.74
		2	4.82 (0.64)	4.91 (0.63)	
		6	5.01 (0.59)^a^	5.12 (0.66)^b^	
	Shin	0	5.01 (0.80)	4.98 (0.91)	0.70
		2	4.76 (0.49)	4.79 (0.50)	
		6	4.85 (0.79)	4.93 (0.82)	

TEWL	Forearm	0	4.60 (2.55)	4.80 (2.95)	0.27
		2	3.70 (3.10)	4.10 (2.15)	
		6	3.00 (3.55)^a^	4.60 (3.40)	
	Abdomen	0	3.90 (2.90)	3.70 (2.45)	0.25
		2	2.40 (3.45)	4.10 (3.60)	
		6	2.70 (2.25)^a^	3.40 (2.95)	
	Shin	0	3.70 (2.30)	3.80 (1.40)	0.23
		2	3.50 (2.25)	3.50 (2.35)	
		6	3.50 (2.85)	4.00 (2.50)	

a *p*-Value <0.05 comparing week 0 to week 6 in pH-modified moisturizer group.

b *p*-Value <0.05 comparing week 0 to week 6 in standard moisturizer group.

**Table 3:** The effect of standard moisturizer versus pH-modified moisturizer on disease severity (SCORAD), DLQI and itch score.

Parameters	Week 0	Week 2	Week 6	*p*-Value
*SCORAD*				
pH-modified moisturizer	14.1 (12.75)	10.5 (13.25)	7 (12.65)	0.00
Standard moisturizer	14.2 (9.1)	10.9 (10.65)	10.5 (11)	0.00
DLQI	8.00 (5.25)	5.00 (7.00)	3.00 (4.25)	0.00
Itch score	4.00 (3.00)	3.00 (3.00)	3.00 (3.25)	0.00

### Standard moisturizer

Similar pattern of reduction in skin pH from week 0 to week 6 with greater reduction at week 2 was observed at standard moisturizer application sites (Table 2). The pH reduction was significant at the forearm, 5.29 (0.94) at week 0; 4.84 (0.55) at week 2 and 5.02 (0.70) at week 6, *p* = 0.00 and the abdomen 5.25 (1.09) at week 0, 4.91(0.63) at week 2, 5.12 (0.66) at week 6, *p* = 0.00. There pH changes at the shin were not significant with 4.98 (0.91) at week 0, 4.79 (0.50) at week 2, and 4.93 (0.82) at week 6, *p* = 0.432. TEWL at the forearms and shins showed non-significant slight reductions at week 2 followed by an increment at week 6 (Table 2). TEWL at the forearm was 4.80 (2.95) at week 0, 4.10 (2.15) at week 2, and 4.60 (3.40) at week 6, *p* = 0.67. At the shins, TEWL was 3.80 (1.40) at week 0, 3.50 (2.35) at week 2 and 4.00 (2.50) at week 6, *p* = 0.913. TEWL increased transiently and later decreased to below baseline values on the abdomen measuring 3.70 (2.45) at week 0, 4.10 (3.60) at week 2 and 3.40 (2.95) at week 6, *p* = 0.80. Results for pH and TEWL at standard moisturizer applications sites are presented in table 2. SCORAD improved significantly with *p* = 0.00, the median value at week 0 was 14.2 (9.1), 10.9 (10.65) at week 2 and 10.5 (11) at week 6 (Table 3).

### pH-modified moisturizer vs standard moisturizer

Greater pH reduction was observed at pH-modified moisturizer sites at the abdomen and shin (Table 2). TEWL values were lower at pH-modified moisturizer sites at forearm and abdomen (Table 2). SCORAD were lower at the pH-modified moisturizer sites at week 2 and week 6 (Table 3). However, there were no statistically significant differences in the pH, TEWL and SCORAD values between the standard moisturizer and pH-modified moisturizer sites. Improvement in SCORAD was mirrored by significant reduction in itch scores and DLQI (Table 3).

One patient developed ipsilateral erythema requiring topical corticosteroid most likely due to a reaction to the pH-modified moisturizer. Two patients developed mild flares which were symmetrical and bilateral. Other side effect reported was hypertrichosis (*n* = 2) over both legs upon completion of the study. Another patient reported mild tingling sensation with pH modified moisturizer application which disappeared after two weeks.

## Discussion

The clinical and biophysical effects of moisturizers on AD skin are under-explored despite extensive use of various types of moisturizers in standard AD management.^14^, ^15^ The effects of mineral oils, a common ingredient in moisturizers on the skin barrier in AD patients are poorly documented. Aqueous cream was chosen as the moisturizer in this study as it is the preferred moisturizer in most of our patients, cost-effective and is more amendable to pH modification. Aqueous cream is preferred by our patients as it feels less greasy, an important factor that facilitate compliance in our tropical hot and humid weather. The aqueous cream did not contain sodium laureth sulfate (SLS), which is the most likely agent responsible for adverse effects reported with aqueous cream.^16^, ^17^, ^18^ Aqueous cream with SLS caused reduced stratum corneum thickness with increase in TEWL after 4 weeks of regular application.^15^, ^16^, ^16^ We observed improvements in both pH and TEWL with aqueous cream (without SLS), pH values were significantly lower from baseline at 6 weeks while there was no significant changes in TEWL. This suggests that the use of SLS rather than aqueous cream should be prohibited for leave-on or wash-off purposes but further confirmatory evidence is required.

Our results showed that skin pH, TEWL and AD severity improved with moisturizer application irrespective of the pH of the moisturizer used. Inclusion of moisturizers in management of AD has been shown to improved disease severity, reduced flares and decreased the amount of topical corticosteroid requirement compared with no moisturizer.^15^ These effects were observed with various types of moisturizers.^15^ The relationship between clinical AD improvement with biophysical parameters like pH, TEWL and hydration are still unclear as these are not evaluated in most moisturizer studies.^14^ Reduction in clinical severity scores seemed to be accompanied by improvement in skin hydration with no change in TEWL.^14^ Data on effect of moisturizers on skin pH is even more lacking. The effect of a moisturizer with pH 4.92 (Diprobase® cream) was compared with aqueous cream (containing SLS) and another moisturizer with pH 7.13 (Doublebase™ gel) in patients with quiescent AD.^12^ Skin pH and hydration increased while TEWL decreased transiently following a single application of all three moisturizers. pH increased significantly with Doublebase™ gel and Diprobase® cream upon 28 days of repeated use, there were no changes in TEWL but hydration was better with Doublebase™ gel. The pH of the moisturizers did not seem to affect skin pH and TEWL differently. The difference in hydration maybe attributed to humectant component of Doublebase™ gel as the other moisturizers contain only occlusive agents. Occlusive dressings have been demonstrated to improve skin hydration, but caused increased pH and TEWL.^19^, ^20^ Long term use of occlusive moisturizers containing paraffin may result in the same effect. The surface pH changes that results from moisturizer application is most likely inadequate to overcome the effects of the skin's intrinsic buffering mechanisms. In addition, concomitant use of skin cleansers and topical steroids which are more alkaline in formulation may have overwhelmed the effect of the moisturizer.

While pH changes with both moisturizers were comparable, we observed significant TEWL improvement at pH-modified moisturizer sites which was not observed at standard moisturizer sites. TEWL reduction is typically not achieved by application of most moisturizers irrespective of its ingredients.^12^, ^14^ TEWL reduction is seen with topical pharmacological interventions such as corticosteroid and calcineurin inhibitor.^21^ The benefit of physiologically compatible pH moisturizer may be due to its effect on TEWL rather than skin surface pH. Acidification of AD skin inhibits activity of pH-sensitive serine proteases that is responsible for accelerated desquamation and increase synthesis of lipids for formation of the extracellular lamellar matrix,^2^ both are key components of the permeability barrier represented by TEWL. Evaluation of stratum corneum compounds is needed to further investigate this aspect.

Both moisturizers were well tolerated, mild and transient adverse effects were observed. All patients have previously used aqueous cream without experiencing adverse effects.

Limitations of this study include the absence of a washout period of the patients’ moisturizer prior to initiation of the study moisturizer. Baseline pH was obtained at least 5 h after the last application of the patients’ routine moisturizer. However, the majority of patients were either moisturizer naïve, using aqueous cream, or aqueous cream in combination with glycerin. In addition, washout was not performed to avoid triggering disease flare. We did not measure skin hydration, thus is unable to show the relationship between hydration and pH. The status of the study participants’ filaggrin deficiency was not determined; this could affect the interpretation of our results.

## Conclusion

Standard aqueous cream and pH-modified aqueous cream both improved skin pH. There were no significant differences in pH and TEWL values between the two moisturizers. pH reduction was observed with both moisturizers. There was significant TEWL improvement with pH-modified moisturizer was not observed with standard moisturizer. Application of both moisturizers resulted in improvement of AD. Moisturizer with physiological pH is useful as an adjunct in the treatment of AD.

## Financial support

Fundamental research grant from 10.13039/501100004515National University of Malaysia, Malaysia.

## Authors' contributions

Goh Siew Wen: Statistic analysis; approval of the final version of the manuscript; conception and planning of the study; elaboration and writing of the manuscript; obtaining, analysis, and interpretation of the data; effective participation in research orientation; intellectual participation in the propaedeutic and/or therapeutic conduct of the studied cases; critical review of the literature; critical review of the manuscript.

Adawiyah Jamil: Approval of the final version of the manuscript; conception and planning of the study; elaboration and writing of the manuscript; obtaining, analysis, and interpretation of the data; effective participation in research orientation; intellectual participation in the propaedeutic and/or therapeutic conduct of the studied cases; critical review of the literature; critical review of the manuscript.

Nazarudin Safian: Statistic analysis; approval of the final version of the manuscript; conception and planning of the study; obtaining, analysis, and interpretation of the data; effective participation in research orientation; intellectual participation in the propaedeutic and/or therapeutic conduct of the studied cases.

Norazirah Md Nor: Statistic analysis; approval of the final version of the manuscript; conception and planning of the study; elaboration and writing of the manuscript; obtaining, analysis, and interpretation of the data; effective participation in research orientation; intellectual participation in the propaedeutic and/or therapeutic conduct of the studied cases; critical review of the manuscript.

Norliza Muhammad: Approval of the final version of the manuscript; conception and planning of the study; elaboration and writing of the manuscript; obtaining, analysis, and interpretation of the data; effective participation in research orientation; intellectual participation in the propaedeutic and/or therapeutic conduct of the studied cases; critical review of the manuscript.

Nur Liyana Saharudin: Approval of the final version of the manuscript; conception and planning of the study; elaboration and writing of the manuscript; effective participation in research orientation; intellectual participation in the propaedeutic and/or therapeutic conduct of the studied cases; critical review of the literature; critical review of the manuscript.

## Conflicts of interest

None declared.

## References

[1] Asher M.I., Montefort S., Björkstén B., Lai C.K., Strachan D.P., Weiland S.K. (2006). Worldwide time trends in the prevalence of symptoms of asthma, allergic rhinoconjunctivitis, and eczema in childhood: ISAAC phases one and three repeat multicountry cross-sectional surveys. Lancet.

[2] Danby S.G., Cork M.J. (2018). pH in atopic dermatitis. Curr Probl Dermatol.

[3] Sparavigna A., Setaro M., Gualandri V. (1999). Cutaneous pH in children affected by atopic dermatitis and in healthy children: a multicenter study. Skin Res Technol.

[4] Hachem J.P., Roelandt T., Schürer N., Pu X., Fluhr J., Giddelo C. (2010). Acute acidification of stratum corneum membrane domains using polyhydroxyl acids improves lipid processing and inhibits degradation of corneodesmosomes. J Invest Dermatol.

[5] Hatano Y., Man M.Q., Uchida Y., Crumrine D., Scharschmidt T.C., Kim E.G. (2009). Maintenance of an acidic stratum corneum prevents emergence of murine atopic dermatitis. J Invest Dermatol.

[6] Lee H.J., Yoon N.Y., Lee N.R., Jung M., Kim D.H., Choi E.H. (2014). Topical acidic cream prevents the development of atopic dermatitis- and asthma-like lesions in murine model. Exp Dermatol.

[7] Hanifin J.M., Rajka G. (1980). Diagnostic features of atopic dermatitis. Acta Dermatol Venereol.

[8] Oranje A.P., Glazenburg E.J., Wolkerstorfer A., de Waard-van der Spek F.B. (2007). Practical issues in interpretation of scoring atopic dermatitis: the SCORAD index, objective SCORAD and the three item severity score. Br J Dermatol.

[9] Finlay A.Y., Khan G.K. (1994). Dermatology Life Quality Index (DLQI) – a simple practical measure for routine clinical use. Clin Exp Dermatol.

[10] Van Scott E.J., Yu R.J. (1974). Control of keratinization with alpha-hydroxy acids and related compounds. Topical treatment of ichthyotic disorders. Arch Dermatol.

[11] Bernstein E.F., Underhill C.B., Lakkakorpi J., Ditre C.M., Uitto J., Yu R.J. (1997). Citric acid increases viable epidermal thickness and glycosaminoglycan content of sun-damaged skin. Dermatol Surg.

[12] Danby S.G., Chalmers J., Brown K., Williams H.C., Cork M.J. (2016). A functional mechanistic study of the effect of emollients on the structure and function of the skin barrier. Br J Dermatol.

[13] Chow S.-C., Shao J., Wang H. (2008). Sample size calculations in clinical research.

[14] Hon K.L., Kung J.S.C., Ng W.G.G., Leung T.F. (2018). Emollient treatment of atopic dermatitis: latest evidence and clinical considerations. Drugs Context.

[15] van Zuuren E.J., Fedorowicz Z., Christensen R., Lavrijsen A., Arents B.W.M. (2017). Emollients and moisturisers for eczema. Cochrane Database Syst Rev.

[16] Tsang M., Guy R.H. (2010). Effect of aqueous cream BP on human stratum corneum in vivo. Br J Dermatol.

[17] Danby S.G., Al-Enezi T., Sultan A., Chittock J., Kennedy K., Cork M.J. (2011). The effect of aqueous cream BP on the skin barrier in volunteers with a previous history of atopic dermatitis. Br J Dermatol.

[18] Mohammed D., Matts P.J., Hadgraft J., Lane M.E. (2011). Influence of aqueous cream BP on corneocyte size, maturity, skin protease activity, protein content and transepidermal water loss. Br J Dermatol.

[19] Hartmann A.A. (1983). Effect of occlusion on resident flora, skin-moisture and skin-pH. Arch Dermatol Res.

[20] Aly R., Shirley C., Cunico B., Maibach H.I. (1978). Effect of prolonged occlusion on the microbial flora, pH, carbon dioxide and transepidermal water loss on human skin. J Invest Dermatol.

[21] Jensen J.M., Pfeiffer S., Witt M., Bräutigam M., Neumann C., Weichenthal M. (2009). Different effects of pimecrolimus and betamethasone on the skin barrier in patients with atopic dermatitis. J Allergy Clin Immunol.

